# Comparison between urine albumin-to-creatinine ratio and urine protein dipstick testing for prevalence and ability to predict the risk for chronic kidney disease in the general population (Iwate-KENCO study): a prospective community-based cohort study

**DOI:** 10.1186/s12882-016-0261-3

**Published:** 2016-05-12

**Authors:** Yorihiko Koeda, Fumitaka Tanaka, Toshie Segawa, Mutsuko Ohta, Masaki Ohsawa, Kozo Tanno, Shinji Makita, Yasuhiro Ishibashi, Kazuyoshi Itai, Shin-ichi Omama, Toshiyuki Onoda, Kiyomi Sakata, Kuniaki Ogasawara, Akira Okayama, Motoyuki Nakamura

**Affiliations:** Division of Cardiology, Department of Internal Medicine, Iwate Medical University, 19-1 Uchimaru, Morioka, Iwate 020-8505 Japan; Iwate Health Service Association, Morioka, Japan; Department of Hygiene and Preventive Medicine, Iwate Medical University, Morioka, Japan; Department of Neurosurgery, Iwate Medical University, Morioka, Japan; The Research Institute of Strategy for Prevention, Tokyo, Japan

**Keywords:** Cardiovascular disease, Renal function, Risk factor, Urine albumin-to-creatinine ratio, Urine dipstick test

## Abstract

**Background:**

This study compared the combination of estimated glomerular filtration rate (eGFR) and urine albumin-to-creatinine ratio (UACR) vs. eGFR and urine protein reagent strip testing to determine chronic kidney disease (CKD) prevalence, and each method’s ability to predict the risk for cardiovascular events in the general Japanese population.

**Methods:**

Baseline data including eGFR, UACR, and urine dipstick tests were obtained from the general population (*n* = 22 975). Dipstick test results (negative, trace, positive) were allocated to three levels of UACR (<30, 30–300, >300), respectively. In accordance with Kidney Disease Improving Global Outcomes CKD prognosis heat mapping, the cohort was classified into four risk grades (green: grade 1; yellow: grade 2; orange: grade 3, red: grade 4) based on baseline eGFR and UACR levels or dipstick tests.

**Results:**

During the mean follow-up period of 5.6 years, 708 new onset cardiovascular events were recorded. For CKD identified by eGFR and dipstick testing (dipstick test ≥ trace and eGFR <60 mL/min/1.73 m^2^), the incidence of CKD was found to be 9 % in the general population. In comparison to non-CKD (grade 1), although cardiovascular risk was significantly higher in risk grades ≥3 (relative risk (RR) = 1.70; 95 % CI: 1.28–2.26), risk predictive ability was not significant in risk grade 2 (*RR* = 1.20; 95 % CI: 0.95–1.52). When CKD was defined by eGFR and UACR (UACR ≥30 mg/g Cr and eGFR <60 mL/min/1.73 m^2^), prevalence was found to be 29 %. Predictive ability in risk grade 2 (*RR* = 1.41; 95 % CI: 1.19–1.66) and risk grade ≥3 (*RR* = 1.76; 95 % CI: 1.37–2.28) were both significantly greater than for non-CKD. Reclassification analysis showed a significant improvement in risk predictive abilities when CKD risk grading was based on UACR rather than on dipstick testing in this population (*p* < 0.001).

**Conclusions:**

Although prevalence of CKD was higher when detected by UACR rather than urine dipstick testing, the predictive ability for cardiovascular events from UACR-based risk grading was superior to that of dipstick-based risk grading in the general population.

## Background

Since the National Kidney Foundation began drawing attention to chronic kidney disease (CKD), several studies have reported that CKD is an independent risk factor for cardiovascular disease and cardiovascular mortality [1, 2]. For people with CKD, the risk for death from a cardiovascular event is up to 20 times greater than the risk for requiring dialysis or transplantation [3]. A recent meta-analysis obtained from 1.5 million inhabitants in mainly Western populations reported that albuminuria levels are important for evaluating overall risk for CKD independent of estimated glomerular filtration rate (eGFR) [4]. In accordance with Kidney Disease Improving Global Outcomes (KDIGO) recommendations, several clinical practice guidelines in Europe, Australia, and Japan have recommended that prognostic grading for CKD should be based on a combination of urine albumin levels and eGFR [5–7].

Measurement of urine albumin, however, is not easily performed in clinical and screening settings, as it is expensive, creates a delay in the availability of results, and must be performed by laboratory technicians. Accordingly, despite relatively limited evidence for its clinical utility, urine dipstick testing remains a popular tool in epidemiological surveys.

To date, few reports have described the relationship between urine albumin-to-creatinine ratio (UACR) and urine protein reagent strip testing in terms of utility for CKD definition and risk grading in the general population. Specifically, it is not yet known how much the characteristics, prevalence, and risk predictive abilities for cardiovascular events of CKD change when UACR is used rather than dipstick testing for detection of this condition. In the current study, we examined the value of using UACR rather than urine dipstick testing to determine CKD prevalence, and compared each method’s ability to predict the risk for cardiovascular events in the general population.

## Methods

### Study participants

This study was a prospective community-based cohort study examining cardiovascular events in Iwate Prefecture in northern Honshu, Japan. A total of 26 469 residents in Ninohe, Kuji, and Miyako consented to participate in the study. All participants provided written informed consent. The study was part of the Iwate-KENCO (Kenpoku Cohort) study, as described previously [8–10].

The baseline survey was conducted between 2002 and 2004 and comprised a self-administered lifestyle questionnaire, blood pressure measurements, anthropometrical measurements, blood collection, and random spot urine sampling. A total of 22 975 participants were enrolled who had complete data for eGFR, UACR, and dipstick urinalysis for proteinuria with no past history of stroke or myocardial infarction (7 841 men and 15 134 women, aged 40–89 years, mean age of 62.9 years).

The study protocol was approved by the Ethics Committee of Iwate Medical University and conducted in accordance with the principles contained in the Declaration of Helsinki.

Blood pressure was measured twice using a Nippon Colin BP-103i II blood pressure monitor (model 513000; Nippon Colin, Komaki, Japan), with the mean value being used for statistical analysis. Peripheral venous blood samples were taken from the upper arm with participants in a seated position after at least 5 min rest. Blood samples were centrifuged immediately after collection and used for measurements. Creatinine (Cr) concentrations in urine and serum were measured by the enzymatic method using a Hitachi 7700 automatic analyzer with standardized calibrators (Hitachi, Tokyo, Japan), and urine albumin measurement was by immunonephelometry (N-antiserum to human albumin assay; Dade Behring, Tokyo, Japan). Urine albumin concentrations were corrected to urine Cr concentrations and expressed as UACR mg/g Cr. Urine protein levels were semi-quantified using a Hema Combistix urine protein reagent testing strip (Siemens Healthcare Diagnostics, Tokyo, Japan). Results of the urine dipstick test were visually interpreted by trained laboratory technicians and recorded as (−), trace, (1+), (2+), or (3+). Data from women who said they were menstruating were excluded in the study, as the urine test might be affected by hemoglobin in urine. eGFR was calculated using the formula devised by the Chronic Kidney Disease Epidemiology Collaboration (CKD-EPI) [11].

**Fig. 1:**
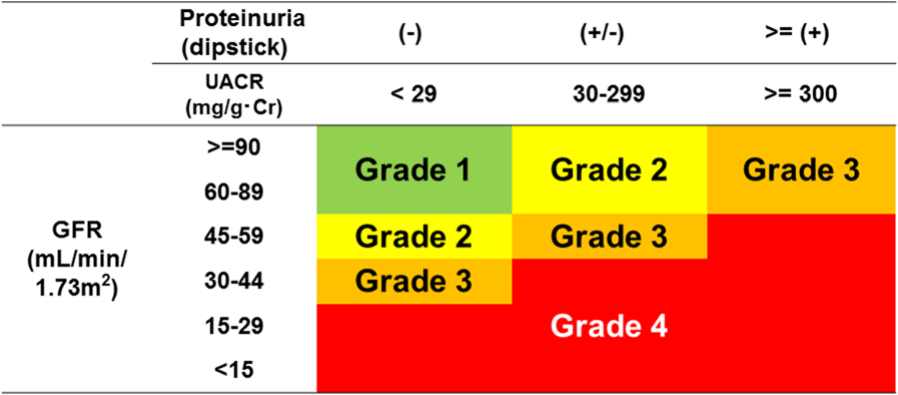
Modified KDIGO risk grading for CKD (six rows × three columns = 18 matrix)

Following practice guidelines developed by the KDIGO proposal [12], participants were divided into an 18-part matrix comprising six categories of eGFR (≥90, 60–89, 45–59, 30–44, 15–29, <15 mL/min/1.73 m^2^) across three categories of UACR (<30, 30–300, >300) or dipstick test (negative, trace, positive) as shown in Fig. 1. The reliability of the allocation of dipstick urinalysis (negative, trace, positive) to three levels of UACR (<30, 30–300, >300) has been reported in previous studies [13, 14]. Accordingly, the definition of CKD was two ways (the dipstick-based definition = dipstick test ≥ trace and eGFR <60 mL/min/1.73 m^2^: the UACR-based definition = UACR ≥30 mg/g Cr and eGFR <60 mL/min/1.73 m^2^) Participants were then classified into four risk grades based on the matrix as shown in the heat map for both definitions (Fig. 1) (green: non-CKD (grade 1); yellow: moderately increased risk (grade 2); orange: high risk (grade 3); red: very high risk (grade 4)).

Diabetes mellitus was defined as a hemoglobin A1c (HbA1c) level ≥6.5 %, fasting blood glucose level ≥126 mg/dL, blood glucose ≥200 mg/dL, or undergoing treatment with antidiabetic drugs including insulin. Hypertension was defined as systolic blood pressure ≥140 mmHg, diastolic blood pressure ≥90 mmHg, or undergoing treatment with antihypertensive drugs. Hyperlipidemia was defined as total cholesterol ≥240 mmHg or undergoing treatment with antihyperlipidemic drugs. Obesity was defined as body mass index (BMI) ≥25.

### Endpoint

The endpoint was determined as the composite of cardiovascular events (stroke, myocardial infarction, and sudden cardiac death occurring within 24 h of the onset of acute symptoms and signs). The incidence of hospitalized myocardial infarction was registered according to criteria of the MONICA study [15]. The study team visited all referral hospitals within the study area and reviewed medical charts or discharge summaries of almost all admitted patients. In cases where clinical data met the definition, the patient’s data were registered. Stroke events were identified by accessing the Iwate Prefecture stroke registration program, which included the entire area where participants lived; details have been described previously [16, 17]. The follow-up survey for acute myocardial infarction and stroke was carried out from the date of the baseline survey until March 2009. The research methods have been described in detail previously [8–10, 17].

### Statistical analysis

Continuous variables are expressed as mean ± SD. Comparisons among CKD risk grades were conducted using one way analysis of variance or chi-squared tests. The Cox proportional hazards model was used to analyze the relative risk (RR) for cardiovascular events in each risk grade adjusted by several cofounding factors (age, sex, hypertension, hyperlipidemia, diabetes mellitus, current smoking, BMI, and atrial fibrillation). To determine concordance between two types of urine protein levels on a dichotomous outcome of UACR (<30, 30–300, >300) and the urine dipstick test (negative, trace, positive), the kappa statistic was calculated. Analyses were performed using the SPSS 20.0 for Windows. Testing the utility of CKD risk grading employing UACR rather than dipstick testing was performed by reclassification tables and tested by Net Reclassification Improvement (NRI) and Integrated Discrimination Improvement (IDI) using the R 3.0.2 software package (www.r-project.org).

## Results

### Clinical characteristics and prevalence

Table 1 shows the clinical backgrounds of the study participants. The average eGFR value for the entire cohort was 76.8 mL/min/1.73 m^2^. Seven hundred and eight participants experienced a first onset of a cardiovascular event during the follow-up period (myocardial infarction or sudden cardiac death: *n* = 145; stroke: *n* = 563).

**Table 1:** Baseline characteristics among CKD risk grades in the general population

	Total (*n* = 22,975)	eGFR + Dipstick			*P* values^a^	eGFR + UACR			*P* values^a^
		Grade 1 (20,902)	Grade 2 (1,462)	Grade 3 & 4 (611)		Grade 1 (16,376)	Grade 2 (5,630)	Grade 3 & 4 (969)	
Age_(year)_	62.9 ± 10.0	62.2 ± 9.8	70.6 ± 8.7	69.7 ± 9.1	< 0.001	61.3 ± 9.8	66.3 ± 9.3	70.6 ± 8.4	< 0.001
Male	34.1 %	32.9 %	45.3 %	49.8 %	< 0.001	33.9 %	33.2 %	43.9 %	< 0.001
BMI_(kg/m_^2^_)_	24.0 ± 3.3	24.0 ± 3.2	24.3 ± 3.4	25.1 ± 3.6	< 0.001	23.8 ± 3.1	24.6 ± 3.5	25.0 ± 3.6	< 0.001
Hypertension	41.5 %	39.4 %	58.1 %	72.7 %	< 0.001	34.4 %	59.6 %	72.5 %	< 0.001
Diabetes Mellitus	6.6 %	6.1 %	8.9 %	19.0 %	< 0.001	4.9 %	9.9 %	16.1 %	< 0.001
Dyslipidemia	16.4 %	16.2 %	18.0 %	18.8 %	0.052	15.7 %	17.7 %	20.3 %	0.052
Current Smoking	12.0 %	12.0 %	11.6 %	14.1 %	0.246	12.3 %	11.0 %	12.1 %	0.246
Atrial Fibrillation	1.4 %	1.1 %	3.8 %	5.7 %	< 0.001	0.8 %	2.6 %	5.2 %	< 0.001
UAC^b^ < 30	75.5 %	79.0 %	52.1 %	10.8 %	< 0.001	100 %	14.1 %	6.4 %	< 0.001
UAC 30-300	22.7 %	20.9 %	42.5 %	39.4 %		0 %	85.9 %	45.2 %	
UAC > 300	1.8 %	0.1 %	5.4 %	49.8 %		0 %	0 %	48.4 %	
Proteinuria (dip stick)									
- Negative (−)	96.4 %	100 %	75.4 %	21.9 %	< 0.001	99.8 %	93.7 %	53.7 %	< 0.001
- Trace (+/−)	1.8 %	0	24.6 %	9.7 %		0.2 %	4.5 %	14.2 %	
- Positive (≥ +)	1.8 %	0	0	68.4 %		0	1.8 %	32.1 %	
eGFR_(mL/min/1.73m_^2^_)_	76.8 ± 10.1	78.5 ± 7.8	60.3 ± 10.8	58.8 ± 18.9	< 0.001	78.8 ± 7.9	74.2 ± 10.7	58.7 ± 16.2	< 0.001
Number of the Events									
- AMI	145	109	22	15	< 0.001	75	51	20	< 0.001
- Stroke	563	455	65	42	< 0.001	286	215	61	< 0.001
- Composite event	708	564	87	57	< 0.001	361	266	81	< 0.001

^a^ Differences among the CKD grades

^b^ Urine Albumin Creatinine Ratio

**Fig. 2:**
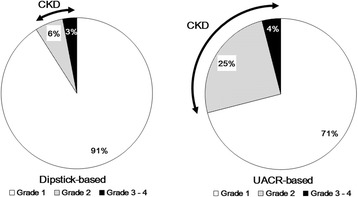
Proportion of CKD risk grades for dipstick- and UACR-based definitions

As shown in Fig. 2 (left), the dipstick-based definition of CKD (dipstick test ≥ trace and eGFR <60 mL/min/1.73 m^2^) showed the prevalence of CKD in the general population as 9 % (men 12 %, women 7 %) and prevalence rates of 91 % for risk grade 1 (green), 6 % for grade 2 (yellow), 2 % for grade 3 (orange), and 1 % for grade 4 (red). As shown in Fig. 2 (right), the UACR-based definition (UACR ≥30 mg/g Cr and eGFR <60 mL/min/1.73 m^2^) showed the prevalence of CKD as 29 % in the general population (men 29 %, women 28 %), with proportions of 71 % in risk grade 1 (green), 25 % in grade 2 (yellow), 3 % in grade 3 (orange), and 1 % in grade 4 (red). The prevalence of CKD according to the UACR-based definition was nearly three times that of the dipstick-based definition (9 % compared with 29 %).

For both methods of CKD assessment, age, BMI, and percentages for diabetes mellitus, hypertension, and atrial fibrillation rose significantly with increasing progression of risk grade, but percentages for hyperlipidemia and current smoking did not differ across risk grades (Table 1).

For dipstick-based risk grades, 60 % of CKD cases showed a negative indication for proteinuria. This result suggests that this method for CKD assessment was mainly based on decreased eGFR (<60 mL/min/1.73 m^2^) with no increase in urine protein levels. In contrast, 79 % of UACR-based CKD cases had elevated UACR levels (≥30 mg/g Cr) without reduced eGFR.

### Relationship between UACR and dipstick testing

**Fig. 3:**
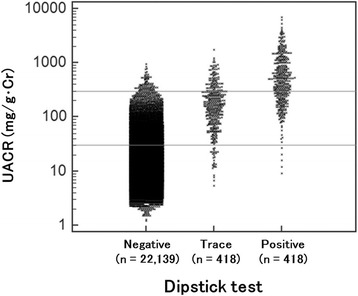
Multiple comparison graph of UACR and dipstick tests

To examine the relationship between UACR levels (<30, 30–300, >300) and semi-quantitative dipstick test results (negative, trace, positive), a multiple comparison graph was constructed (Fig. 3). Even when dipstick urinalysis was negative, 22 % of participants had UACR ≥30 mg/g Cr. When the degree of coincidence between UACR levels (<30, 30–300, >300) and dipstick results (negative, trace, positive) was tested using the kappa statistic, the coefficient was relatively low (0.171). Sensitivity of the urine dipstick test (≥ + 1) for UACR (more than 30 mg/g Cr) was 8 %, and specificity was 100 %.

### Risk grades and cardiovascular events

**Fig. 4:**
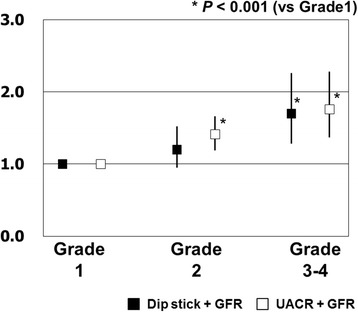
Relative risks and 95 % CI for cardiovascular events for two types of CKD risk grades

When compared with the non-CKD group (risk grade 1), RR for cardiovascular events derived from a Cox proportional hazards model was 1.36 (95 % CI: 1.12–1.65) for dipstick-based CKD, and 1.47 (95 % CI: 1.25–1.72) for UACR-based CKD. Both methods of CKD assessment on the whole showed similar predictive abilities for cardiovascular events. When the cohort was divided into four risk grades based on urine dipstick testing, RR for cardiovascular events were 1.20 (95 % CI: 0.95–1.52) for grade 2 and 1.70 (95 % CI: 1.28–2.26) for grade 3 and above, showing no significant increase in RR in grade 2 (Fig. 4). On the other hand, for UACR-based grades, there was a significant increase in RR for cardiovascular events in both grade 2 (RR: 1.41; 95 % CI: 1.19–1.66) and grade 3 and above (RR: 1.76; 95 % CI: 1.37–2.28).

### Reclassification analysis

**Fig. 5:**
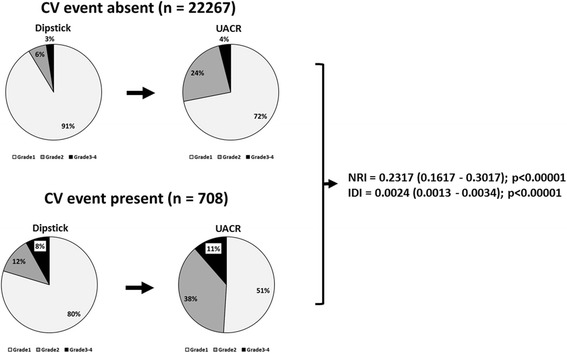
Reclassification analysis. Changes in risk stratification capacity derived from reclassification tables in terms of dipstick-based CKD risk grades vs. UACR-based CKD risk grades. For comparison with the dipstick-based model, the percentage of risk grades 3–4 in the events group increased (from 20 to 49 %) in the UACR-based model. NRI: Net Reclassification Improvement; IDI: Integrated Discrimination Improvement

Risk stratification capacities for cardiovascular events for dipstick-based risk grades (low risk (grade 1), intermediate risk (grade 2), high risk (grades 3 and 4)) vs. UACR-based grades derived from the reclassification table are shown in Fig. 5. In the events group (*n* = 708), 80 % were categorized as low risk by the dipstick-based model, compared with 51 % by the UACR-based risk grade. In addition, according to dipstick-based CKD classifications, prevalence of CKD was only 20 % in the event group (grade ≥ 2), whereas with UACR-based CKD classification, prevalence of CKD was 49 %.

These results suggest that when the UACR level was used rather than dipstick testing for heat map risk grading, overall predictive ability for cardiovascular events significantly improved (NRI = 0.232, *p* < 0.001; IDI = 0.0024, *p* < 0.001).

## Discussion

Results of this study suggest that the cardiovascular predictive performance for CKD risk grading based on UACR plus reduced eGFR (UACR-based risk grading) was superior to that of dipstick testing plus reduced eGFR (dipstick-based risk grading) in the general population.

In addition to generating a high degree of risk for the development of end-stage renal disease, CKD is also an important risk factor for cardiovascular events such as atherosclerotic coronary heart disease, stroke, and all-cause mortality [1, 2, 4]. CKD is therefore a more important risk factor for cardiovascular disease than diabetes [18]. Several studies have reported that the number of patients experiencing cardiovascular events and cardiovascular death is greater than the number receiving dialysis [1, 3]. It has been determined that the coexistence of CKD in patients with cardiac disorders is one of the important independent prognostic markers [19, 20]. In view of this, it is recommended that patients at high risk of a cardiovascular event should have the risk managed early on, and should undergo screening to determine both GFR and urine albumin [21].

UACR measurement is somewhat costly compared with dipstick urinalysis, especially in mass screening settings. Therefore, although dipstick testing has low sensitivity for renal dysfunction [22], this form of testing is frequently used in general population studies because of its simplicity and low cost. Few studies have reported on the relationship between UACR and dipstick testing in large-scale general population samples of more than 20 000 participants. Konta et al. reported that, in the general population (*n* = ~2 300), cases with negative dipstick test results sometimes showed UACR ≥30 mg/g Cr, and that even trace levels of proteinuria detected by dipstick testing often showed UACR ≥300 mg/g Cr in diabetic or elderly patients [13]. Indeed, the degree of coincidence between UACR and dipstick testing was suboptimal in the current study’s population (Fig. 3).

To the best of the authors’ knowledge, no reports have directly compared the two methodologies (UACR-based risk grading or dipstick-based risk grading) to determine the better cardiovascular risk predictor in screening settings for the general population. The current study suggests that risk grading based on UACR is superior for predicting the development of cardiovascular events compared with dipstick-based grading (Fig. 5).

Prevalence of UACR >30 mg/g Cr was found to be 25 % in the current study, a rate clearly higher than those found in populations in North America (NHANES III: 9 %) [23], Europe (PREVEND study: 7 %) [24], and Australia (AusDiab Kidney study: 6 %) [25]. The reason for the high prevalence of significant albuminuria in our population remains unknown. It may be, however, because of the higher age of our study cohort, as participants under 40 years of age were excluded. The cohort also included a high proportion of women (65 %). As women excrete less Cr per day than men, the urine albumin concentration corrected to the urine Cr concentration may have been calculated to be relatively high, and therefore easily exceeded the threshold of microalbuminuria of >30 mg/g Cr. It is possible that these factors contributed to the relatively high prevalence of UACR-based CKD in women in the current cohort.

Given the high prevalence of microalbuminuria in this cohort, the high frequency of CKD was mainly because of the high incidence of UACR ≥30 mg/g Cr. Few reports directly describe the prevalence of CKD using UACR combined with the modern eGFR calculation (CKD-EPI) in a large number of participants. Therefore, the true prevalence of CKD as defined by UACR levels and reduced eGFR within the general population remains unclear. It seems unlikely that the high prevalence of CKD would be specific to the current population. Yamamoto et al. reported that 24 % of patients with a negative dipstick test showed elevated UACR ≥30 mg/g Cr [26], which is almost the same as the value seen in the current study (22 %). Terawaki et al. reported that of the 241 159 participants who received specific health examinations (mean age: 63 years, 39 % men), mean eGFR was 76 mL/min/1.73 m^2^ [27], which again is almost the same as the current study. It therefore appears unlikely that renal function in the current cohort differs from that in populations from other regions of Japan.

Under the Japanese health insurance system, routine measurement of urine albumin is permitted for patients at high risk for cardiovascular disorders such as diabetes mellitus but not for other disorders. Microalbuminuria has been reported to be a risk factor for cardiovascular disease among non-diabetic and non-hypertensive patients [28]. It is possible that for patients with diabetes, hypertension, and obesity, cardiovascular risk assessment including albumin measurement for identification of CKD and more strict management of underlying disorders may improve prognosis. Hence, it may be beneficial to measure UACR to detect CKD even in populations at moderate cardiovascular risk, specifically diabetic, hypertensive, and obese people. As urine albumin measurement is relatively expensive compared with the cost of dipstick testing, further studies are required to identify the cost effectiveness of CKD screening using UACR for reducing cardiovascular events and mortality.

The current study has several strengths. To the best of the authors’ knowledge, this study included the largest general population sample in whom UACR and urine dipstick testing has been performed. UACR was measured in fresh urine samples without long term freezing and repeated thawing. Cardiovascular events were captured prospectively according to previously determined standard epidemiological criteria and confirmed by research staff on medical chart reviews. Baseline data including clinical characteristics and biochemical data were determined well before the start of the follow-up study. However, despite the study’s merits, some limitations must be considered when interpreting the results. First, measurement of urine albumin was based on a single measurement, which did not completely comply with the clinical requirement for multiple urine collections for establishing a diagnosis of CKD. Second, what effects prescribed drugs such as renin-angiotensin inhibitors have on cardiovascular events and urine albumin levels in the current population are not known. Third, the study employed only the CKD epidemiology collaboration (CKD-EPI) equation for eGFR and did not use other formulae, since there is no consensus on the best equation for eGFR in Asian populations.

## Conclusion

Although the prevalence of CKD in the general population was found to be higher when determined by eGFR and UACR compared with urine protein dipstick testing, the predictive ability for future cardiovascular events from UACR-based risk grading was superior to that of dipstick-based risk grading. Further studies including cost effectiveness analysis are needed to determine how best to utilize urine albumin measurement rather than dipstick testing to reduce the risk for cardiovascular events in mass screening settings.

### Availability of supporting data

All data supporting the study is presented in the manuscript or available upon request from the corresponding author of this manuscript, Y Koeda.

### Ethics approval and consent to participate

The study protocol was approved by the Ethics Committee of Iwate Medical University and conducted in accordance with the principles contained in the Declaration of Helsinki. All participants provided written informed consent.

## Abbreviations

BMI: body mass index
CKD: chronic kidney disease
CKD-EPI: Chronic Kidney Disease Epidemiology Collaboration
Cr: creatinine
HbA1c: hemoglobin A1c
IDI: Integrated Discrimination Improvement
KDIGO: Kidney Disease Improving Global Outcomes
NRI: Net Reclassification Improvement
RR: relative risk
UACR: urine albumin-creatinine ratio
